# When Radiation Therapy Becomes a Foe: A Rare Case of Radiation-Induced Angiosarcoma of Head and Neck

**DOI:** 10.7759/cureus.35122

**Published:** 2023-02-17

**Authors:** Jay Vakil, Maria Cristina Cuartas-Mesa, Liu Jian Hua, Vaishali Deenadayalan, Ekrem Turk

**Affiliations:** 1 Internal Medicine, John H. Stroger, Jr. Hospital of Cook County, Chicago, USA; 2 Pathology, University of Illinois Chicago, Chicago, USA

**Keywords:** chemo-radiation, cancer radiotherapy, hematology-oncology, radiation and clinical oncology, radiation oncology education, head and neck neoplasms

## Abstract

Angiosarcomas are a rare subtype of sarcomas originating from vascular endothelial cells. Though frequently found in the head and neck area, there are minimal reports of radiation-induced angiosarcomas in this area. They have a poor prognosis due to a high rate of lymph node metastasis and, hence, require to be addressed promptly in order to improve survival. We present a rare case of radiation-induced angiosarcoma in a patient previously irradiated for squamous cell carcinoma of the neck. Due to variable and complex patient presentations of the disease, this case will help raise awareness of an uncommon complication of a common treatment offered to patients.

## Introduction

Radiation therapy is well known to be an effective treatment for various types of cancer, and it is used with curative and palliative intent. However, it also has severe and adverse effects secondary to DNA breakdown, including the risk of developing secondary cancer, usually occurring in two waves after exposure, the first around five years and the second around 10 years. The two most common types are squamous cell carcinoma and sarcomas [1]. 

The prevalence of sarcomas accounts for about 0.1% of head and neck cancers [2]. The most frequent histology is osteosarcomas and fibrosarcomas [3]. Angiosarcomas, on the other hand, are rare and have a poor prognosis, given their high rate of lymph node metastasis and median five-year survival of 26-51% [4]. For this reason, they need to be identified promptly to improve survival. Clinical suspicion is complex as its presentation varies widely and can sometimes resemble a benign pathology [2]. We present a case of radiation-induced angiosarcoma of the head and neck, a rare but potentially lethal complication of radiation treatment that will help raise awareness and allow practitioners to take into consideration when offering treatment.

## Case presentation

A 60-year-old female presented in August 2013 with a worsening left-sided neck mass for several months. Physical exam revealed multiple, firm, immobile lymph nodes on the left side of her neck. Core lymph node biopsy revealed metastatic squamous cell carcinoma. Upon completion of workup, she was found to have multiple positive lymph nodes with extracapsular spread, but no primary could be identified. She was diagnosed with Stage 4 TxN2bM0 squamous cell carcinoma of the head and neck with unknown primary. Following direct laryngoscopy with multiple random biopsies, bilateral tonsillectomy, and left neck dissection, a primary site could still not be identified. She completed adjuvant cisplatin chemotherapy treatment and 66 Gy RT to high-risk areas in 33 fractions in March 2014.

**Figure 1:**
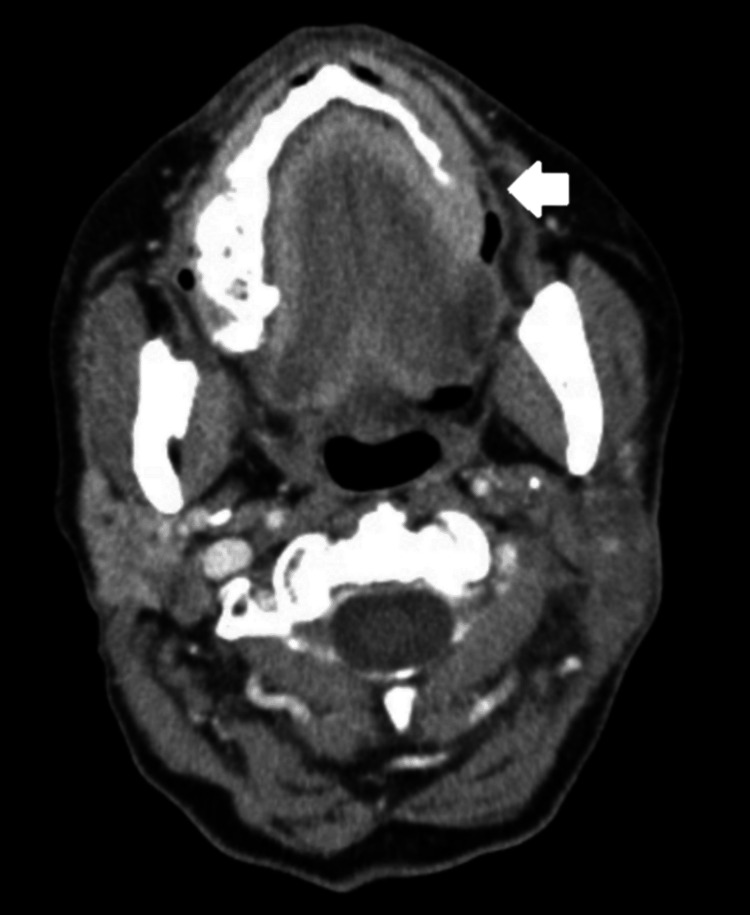
CT neck soft tissue with IV contrast Long-standing postoperative changes from a left neck dissection (white arrow)

**Figure 2:**
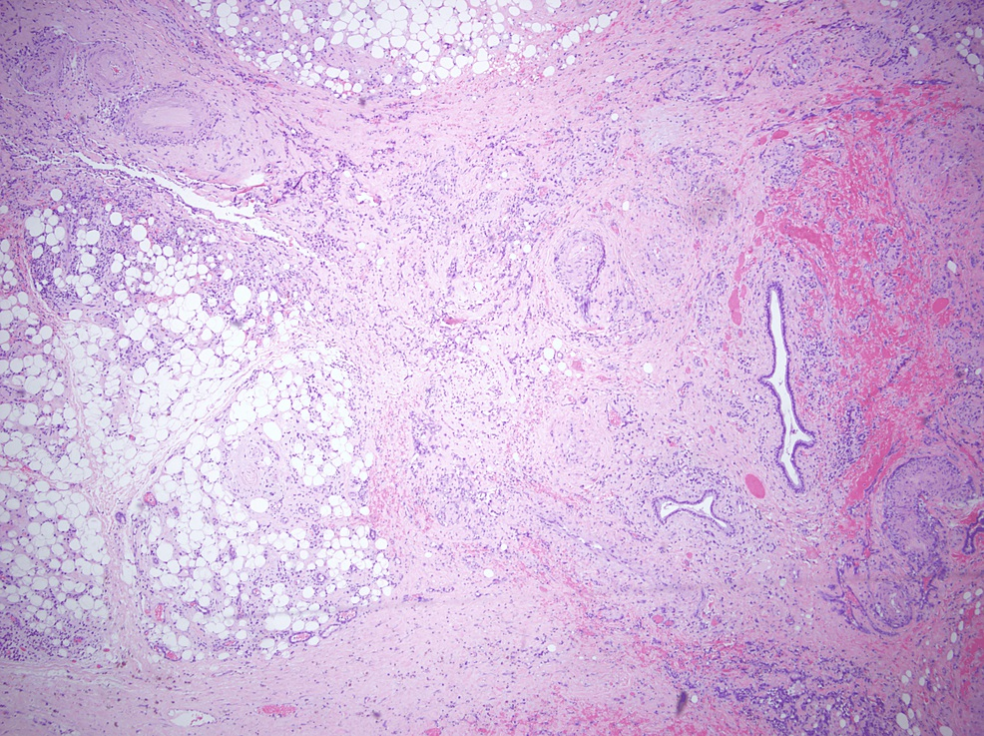
Core needle biopsy (Slide 1) Detached epidermis with underlying subcutaneous tissue involved by vasoformative mesenchymal neoplasm with significant nuclear atypia

**Figure 3:**
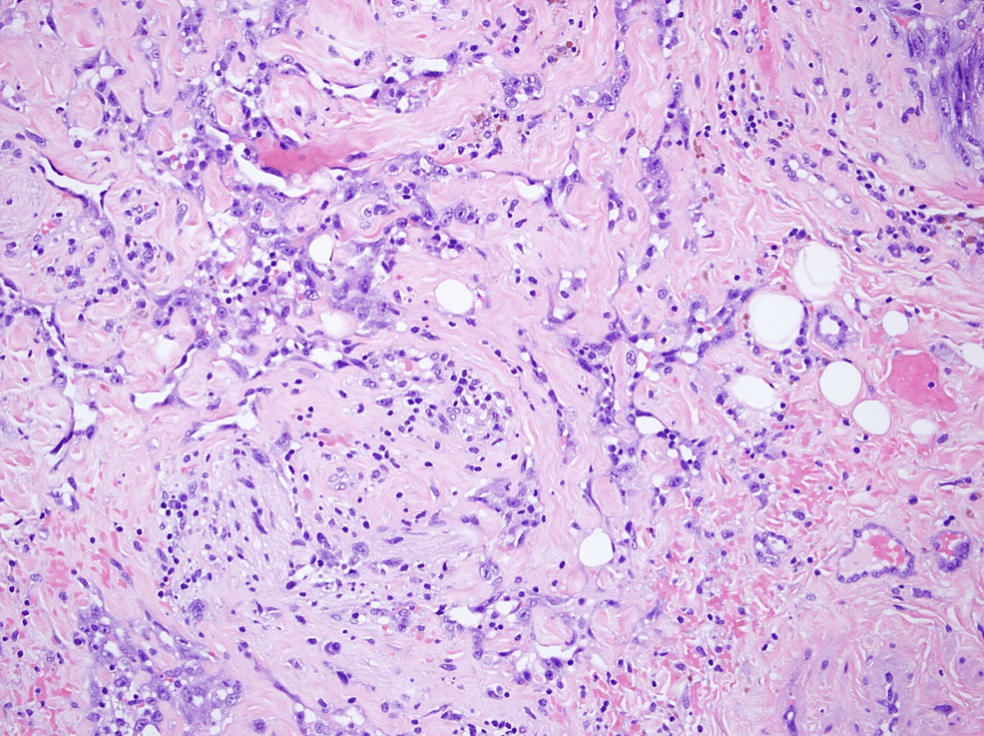
Core needle biopsy (Slide 2) There is a high-grade malignancy positive for CD31, CD34, and MYC. D2-40 is focally positive. The tumor cells are negative for keratins AE1/AE3, HHV8, and p40.

In March of 2021, the patient started to complain of a left-sided neck mass with associated pain that was present ever since her procedure in 2013. CT imaging revealed postoperative and post-treatment changes in the left neck (Figure 1). In addition, two foci of enhancement involving the skin and subcutaneous soft tissues were noted overlying the left hemimandible. A core needle biopsy of the lesion showed radiation-induced angiosarcoma (Figures 2, 3). She underwent two wide local excisions, left radial forearm free flap surgery, and left upper extremity skin graft in July 2021 but her course was complicated by graft failure and positive surgical margins with perineural invasion. Soon after, the patient started to develop worsening dysphagia with esophagogastroduodenoscopy (EGD) results showing extrinsic compression due to an unclear cause. In October 2021, the patient was found to have a positive arytenoid biopsy for angiosarcoma on laryngoscopy. She was found to have erythematous supraglottitis and had a tracheostomy placed in November 2021. The patient decided she did not want further cancer-directed care and agreed to pursue comfort care measures in December 2021. 

## Discussion

Angiosarcomas are a rare subtype of sarcomas that originate from vascular endothelial cells [5]. As they emerge in the vasculature, they can develop in any organ. Two strong risk factors linked to the development of angiosarcoma included chronic lymphedema and prior radiation therapy, as seen in our patient. In addition, they more commonly affect the elderly and are twice as likely to occur in males than females [6]. Frequently noted locations include cutaneous scalp lesions in elderly white men as well as in cancer locations treated with radiation, the most notable being breast cancer and lymphomas [7]. Angiosarcomas are extremely rare, representing less than 1% of soft tissue sarcomas [5]. Furthermore, though angiosarcomas are frequently found in the head and neck, there are minimal reports of radiation-induced angiosarcomas in this area. 

Presentations of patients with radiation-induced sarcomas may vary but include bruise-like lesions, dusky plaques, chronic edema or cellulitis, ulcerated nodules, or pyoderma [8]. Providers should have increased suspicion if a patient develops skin lesions in an area that has been previously irradiated, even several years prior. 

The exact mechanism leading to the development of radiation-induced angiosarcoma isn’t well known though it is suspected that previous radiation exposure leads to damage to DNA within cells [3]. The microscopic cytological smears show spindle-shaped sarcomatous cells, numerous naked nuclei, and a hemorrhagic background [9]. Histological sections show typical angiosarcoma, rich in vascular spaces surrounded by spindle-shaped sarcoma cells. 

The overall prognosis of radiation-induced sarcoma in general is poor [10]. They are aggressive in nature and have poor chemo and radio sensitivity, making surgery the most effective treatment option for patient survival. Furthermore, achieving negative surgical margins can be difficult due to the underlying fibrotic changes secondary to radiation. In a single-institution review of patients with radiation-induced sarcomas, 22 patients were evaluated and found to have a five-year survival of 14.4% [10]. Further evaluations of only the subtype of angiosarcoma have not been done in this region of the body. 

## Conclusions

Radiation-induced angiosarcoma, though commonly reported in the breast, is rare in the head and neck area, with very few cases reported. Although treatment of choice is surgery, negative surgical margins are difficulty to obtain due to underlying fibrotic changes secondary to radiation, as seen in our patient. Our patient was diagnosed with her second malignancy 7 years after the first. This highlights the need for practitioners to keep close evaluation and prompt intervention for patients who have previously received radiation therapy, a common treatment modality for patients with malignancies.
